# Whole genome sequencing of consanguineous families reveals novel pathogenic variants in intellectual disability

**DOI:** 10.1111/cge.13470

**Published:** 2018-12-07

**Authors:** Ann‐Charlotte Thuresson, Cecilia Soussi Zander, Jin J. Zhao, Jonatan Halvardson, Khurram Maqbool, Else Månsson, Eric Stenninger, Ulrika Holmlund, Ylva Öhrner, Lars Feuk

**Affiliations:** ^1^ Department of Immunology, Genetics and Pathology, Science for Life Laboratory Uppsala University Uppsala Sweden; ^2^ Department of Pediatrics Örebro University Hospital Örebro Sweden; ^3^ Department of Pediatrics Västerås Hospital Västerås Sweden

## Abstract

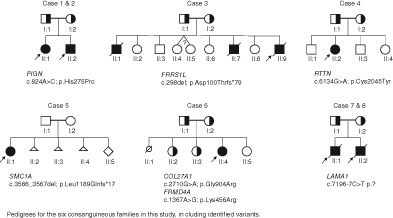

*To the Editor*:

Despite recent progress in identification of recessive intellectual disability (ID) genes, many recessive ID genes remain to be identified. In this study we aimed to identify pathogenic variants in families that had previously gone through whole exome sequencing (WES) and chromosomal microarray analysis.^1^ Six consanguineous families (4 trios and 2 quads) were selected for whole genome sequencing (WGS), where the proband had an ID or developmental delay (DD) diagnosis in combination with dysmorphology/congenital malformations. WGS was performed to 30× coverage with Illumina HiSeqX using standard protocols. Sequence reads were mapped to human reference genome Hg19. Variants were called and filtered as previously described,^1^ followed by structural variation analysis. Variants of interest were verified by Sanger sequencing.

Seven novel variants were identified in the six families (Table 1). Six of the variants showed recessive inheritance, that is, homozygous in the patients and inherited from heterozygous parents, and a de novo deletion was identified in a female patient in the X‐chromosome gene *SMC1A*. Five of the genes *PIGN, FRRS1L*, *RTTN, SMC1A* and *LAMA1* cause disorders associated with ID/DD. Although the variants in *RTTN* and *LAMA1* are candidates, symptoms of the probands matched the expected phenotypes for these disorders.

**Table 1:** Variants classified as pathogenic, likely pathogenic or of uncertain significance in this study, including CADD score, MAF, associated disorder including OMIM number and phenotype of the patients

Family ID	Case	Gender	Gene (Transcript)	Variant	Zygosity	CADD score	MAF^a^	Classi‐fication	Associated disorder (OMIM designation and number)	Type	Phenotype	Appearance
1	1	F	*PIGN* (NM_176787.4)	c.824A > C (p.His275Pro)	Homozygous	25.8	—	LP	Multiple congenital anomalies‐hypotonia‐seizures syndrome 1 (MIM #614080)	AR	Severe ID (no social interaction or speech), severe hypotonia, epilepsy/focal seizures. Thin corpus callossum, cerebellar atrophy, increased cerebrospinal fluid spaces, visual impairment (pathological VEP), PEG feeding. Normal serum alkaline phosphatase levels.	Microcephaly (OFC −4SD), round face, low anterior hairline, broad nasal bridge, high‐arched palate, small chin, slender feet and hirsutism. Scoliosis. Dry skin, peeling on feet.
1	2	M	*PIGN* (NM_176787.4)	c.824A > C (p.His275Pro)	Homozygous	25.8	—	LP	Multiple congenital anomalies‐hypotonia‐seizures syndrome 1 (MIM #614080)	AR	Severe ID (no social interaction or speech), severe hypotonia, epilepsy/focal seizures. Thin corpus callossum, PEG feeding. Normal serum alkaline phosphatase levels.	Bitemporal narrowing, broad nasal bridge, large ears, thin upper‐vermillion and a smooth, long philtrum. Scoliosis. Dry skin, peeling on feet.
2	3	M	*FRRS1L* (NM_014334.3)	c.298del (p.Asp100Thrfs*79)	Homozygous	25.8	—	P	Epileptic encephalopathy, early infantile, 37 (MIM#616981)	AR	Severe ID (absent speech), psychomotor regression, severe hypotonia, infantile spasms, severe epilepsy, myclonus of facial muscles and upper limbs. Frontotemporal cerebral atrophy, neurodegeneration. Myopia. PEG feeding, osteopenia. Died at age 5 y and 9 m.	Full cheeks, gingival overgrowth, thin calf muscles, hypermobility/joint laxity in hands, knees and feet, coxa vara.
3	4	F	*RTTN* (NM_173630.3)	c.6134G > A (p.Cys2045Tyr)	Homozygous	32	—	VUS	Microcephaly, short stature, and polymicrogyria with seizures (MIM #614833)	AR	Moderate ID, IUGR, reduced fetal movements, respiratory problems in infacy, necrotizing enterocolitis, perimyocarditis, recurrent infections. Restrictive cardiomyopathy, chronic heart insufficiency, hypothyroidism.	Microcephaly (OFC > −4SD), growth retardation (length −6SD/weight −2SD). Thick eyebrows. Long and thin fingers, bruising susceptibility.
4	5	F	*SMC1A* (NM_006306.3)	c.3566_3567del (p.Leu1189Glnfs*17)	Heterozygous	35	—	P	Cornelia de Lange syndrome 2 (MIM #300490)	XD	ID, motor delay, speech delay, seizures from 2 y. Gastroesophageal reflux first 6 m. Heart murmurs.	Flat face, arched eyebrows, slight synophrys, hypertelorism, low‐set ears, short columella, short and smooth philtrum, thin upper vermillion, underbite, crowded teeth, hypermobility of distal interphalangeal joints, proximally placed thumbs. Short thorax, slight kyphoscoliosis, widely spaced nipples. Partial syndactyly dig 2‐3 of feet, bilateral clinodactyly. Hirsutism. Height −2.5SD and weight −2SD.
5	6	F	*COL27A1* (NM_032888.3)	c.2710G > A (p.Gly904Arg)	Homozygous	27.6	—	LP	Steel Syndrome (MIM#615155)	AR	Mild ID, SGA, delayed myelination, congenital hip dislocation, tethered spinal cord, femoral collum pseudoarthrosis. Bilateral hearing impairment,	Growth retardation, hypertelorism, widely spaced nipples, sacral dimple, thoracal scoliosis, short upper limbs, coxa vara and pes cavus.
			*FRMD4A* (NM_018027.4)	c.1367A > G (p.Lys456Arg)	Homozygous	23.1	7.953e‐6	VUS	Agenesis of the corpus callosum with facial anomalies and cerebellar ataxia (MIM #616819)	AR		
6	7	M	*LAMA1* (NM_0005559.3)	c.7196‐7C > T (p.?)	Homozygous	5.2	1.698e‐4	VUS	Poretti‐Boltshauser syndrome (MIM#615960)	AR	Severe DD, no movements, absent speech, severe epilepsia, sensorineural hearing impairment, visual impairment with opticus atrophy. Subdural hemorrhage, ventriculomegaly, reduced volume of the brain. Died at age 1.5 y	Microcephaly
6	8	M	*LAMA1* (NM_0005559.3)	c.7196‐7C > T (p.?)	Homozygous	5.2	1.698e‐4	VUS	Poretti‐Boltshauser syndrome (MIM#615960)	AR	Profound ID, no movements, absent speech, severe epilepsia, sensorineural hearing impairment, visual impairment with opticus atrophy. Hypothyreosis, hypercortisolism. Died at age 7.5 y.	Microcephaly, severe kyphoscoliosis, contractures of elbows, hands, knees and feet.

Abbreviations: AR, autosomal recessive; DD, developmental delay; ID, intellectual disability; IUGR, intrauterin growth retardation; LP, likely pathogenic; m, months; MAF, minor allele frequency; OFC, occipitofrontal circumference; P, pathogenic; SD, standard deviation; SGA, small for gestational age; VUS, variant of uncertain significance; XD, X‐linked dominant; y, years.

a MAF reported amongst the >141 000 exom/genome sequences included in the gnomAD database. No identified homzygotes in gnomAD.

Phenotypes not previously described were detected in some probands. Case 4, with a bi‐allellic variant in *RTTN*, presented with cardiomyopathy, but not the previously described brain anomalies. The contribution of other variants causing the cardiomyopathy cannot be excluded.

In‐frame and missense variation in *SMC1A* causes Cornelia de Lange syndrome (CdLS), whereas truncating variants are associated with a seizure disorder lacking the characteristic facial features of CdLS.^2^ Case 5 with a truncating frame‐shift variant presented with seizures from age 25 months, ID, and mild facial dysmorphology. The developmental impairment in our patient seems to be milder compared to the patients in previous studies.^2^

Moreover, we provide supporting evidence of extending the phenotype of Steel syndrome, caused by pathogenic variants in *COL27A1*, to include ID and hearing impairment (case 6). ID has previously been reported in one case,^3^ and hearing impairment in two cases.^3^, ^4^

*FRMD4A* is a candidate ID gene reported in only a single large pedigree, with a homozygous frame‐shift variant.^5^ The homozygous missense variant identified in *FRMD4A* (case 6) does not have any of the previously described features apart from ID, which is mild in our case. One explanation could be that missense variations in *FRMD4A* will give rise to an alternative phenotype, potentially supported by the ExAC *z*‐score for missense variants (*z* = 3.23). Nevertheless, we cannot rule out the contribution of *FRMD4A* to ID in our patient.

Six of seven identified variants resided within coding sequence and yet they were not identified using WES. Sequence capture required in WES leads to uneven coverage of exonic regions, compared to WGS. Low coverage may lead to heterozygous sites being called as homozygous, which may hamper analysis in recessive families where filtering is based on both parents being heterozygous carriers. The de novo deletion in *SMC1A* was not detected with WES despite adequate coverage across the exon, potentially caused by the deletion causing allele‐specific target capture. Variants might also be missed if WES used an early version of the capture kit or if the gene was not disease‐associated at the time of analysis.

In summary, we provide supporting evidence of extending the phenotype of Steel syndrome, to also include hearing impairment and ID. This study shows that WGS is a highly efficient strategy to provide a molecular diagnosis for ID in consanguineous families and gave a high diagnostic yield in families where previous WES failed to yield a diagnosis.
